# Knockdown of CDK2AP1 in human embryonic stem cells reduces the threshold of differentiation

**DOI:** 10.1371/journal.pone.0196817

**Published:** 2018-05-07

**Authors:** Khaled N. Alsayegh, Steven D. Sheridan, Shilpa Iyer, Raj Raghavendra Rao

**Affiliations:** 1 Department of Human and Molecular Genetics, School of Medicine, Virginia Commonwealth University, Richmond, VA, United States of America; 2 King Abdullah International Medical Research Center, King Saud bin Abdulaziz University for Health Sciences, Jeddah, Saudi Arabia; 3 Center for Genomic Medicine, Massachusetts General Hospital, Harvard Medical School, Boston, MA, United States of America; 4 Department of Biological Sciences, Fulbright College of Arts and Sciences, University of Arkansas, Fayetteville, AR, United States of America; 5 Department of Biomedical Engineering, College of Engineering, University of Arkansas, Fayetteville, AR, United States of America; Macau University of Science and Technology, MACAO

## Abstract

Recent studies have suggested a role for the Cyclin Dependent Kinase-2 Associated Protein 1 (CDK2AP1) in stem cell differentiation and self-renewal. In studies with mouse embryonic stem cells (mESCs) derived from generated mice embryos with targeted deletion of the Cdk2ap1 gene, CDK2AP1 was shown to be required for epigenetic silencing of Oct4 during differentiation, with deletion resulting in persistent self-renewal and reduced differentiation potential. Differentiation capacity was restored in these cells following the introduction of a non-phosphorylatible form of the retinoblastoma protein (pRb) or exogenous Cdk2ap1. In this study, we investigated the role of CDK2AP1 in human embryonic stem cells (hESCs). Using a shRNA to reduce its expression in hESCs, we found that CDK2AP1 knockdown resulted in a significant reduction in the expression of the pluripotency genes, OCT4 and NANOG. We also found that CDK2AP1 knockdown increased the number of embryoid bodies (EBs) formed when differentiation was induced. In addition, the generated EBs had significantly higher expression of markers of all three germ layers, indicating that CDK2AP1 knockdown enhanced differentiation. CDK2AP1 knockdown also resulted in reduced proliferation and reduced the percentage of cells in the S phase and increased cells in the G2/M phase of the cell cycle. Further investigation revealed that a higher level of p53 protein was present in the CDK2AP1 knockdown hESCs. In hESCs in which p53 and CDK2AP1 were simultaneously downregulated, OCT4 and NANOG expression was not affected and percentage of cells in the S phase of the cell cycle was not reduced. Taken together, our results indicate that the knockdown of CDK2AP1 in hESCs results in increased p53 and enhances differentiation and favors it over a self-renewal fate.

## Introduction

CDK2AP1 (Cyclin Dependent Kinase-2 Associated Protein-1) has lately gained importance in the field of stem cell research, with initial studies identifying it as one of the stem cell-specific genes that are enriched in both embryonic and adult stem cells [1–4]. It has also been identified as one of many genes that are expressed in early stage preimplantation embryos [4,5]. In studies conducted with homozygous Cdk2ap1 knockout mESCs, the effects of LIF (leukemia Inhibitory Factor) removal on the Cdk2ap1 knockout and wild type cells were examined [6]. Upon the removal of LIF, Cdk2ap1+/+ mESCs showed signs of differentiation and reduced the expression of the pluripotency gene Oct4, whereas the Cdk2ap1-/- mESCs maintained their expression of Oct4 and did not display any signs of differentiation. Further investigation revealed that deletion of Cdk2ap1 in mESCs prevented methylation of the Oct4 promoter which resulted in the maintenance of its expression even in 8 day old embryoid bodies [6,7]. In a subsequent study, deletion of Cdk2ap1 in mESCs prevented differentiation and resulted in persistent self-renewal, attributed to hyper-phosphorylation of the retinoblastoma protein (pRb) [8]. Differentiation capacity in the Cdk2ap1-/- mESCs was restored upon the introduction of a mutant non-phosphorylatable form of pRb or exogenous Cdk2ap1[8]. Taken together, these studies suggest that deletion of Cdk2ap1 in mESCs results in increased self-renewal and a decrease in differentiation. In this study, we have uncovered a novel function for CDK2AP1 in the self-renewal and pluripotency of human embryonic stem cells. Knockdown of CDK2AP1 in hESCs most likely results in increased p53 and enhances differentiation potential and favors it over self-renewal.

## Materials and methods

### Generation of inactivated feeder layers and conditioned medium

Mouse embryonic fibroblasts (MEFs) were isolated from embryos derived from 13.5 days pregnant CF1 mice and maintained in MEF-medium containing Dulbecco’s modified Eagle’s medium (DMEM) with 4.5 g/L glucose, 2 mM L-glutamine, 1% penicillin/streptomycin, and 10% fetal bovine serum. MEF-conditioned medium (CM) was generated by seeding 1x 10^6^ mitotically inactivated MEFs on 10 cm dishes in 10 ml MEF- medium. The following day, MEF-medium was replaced with 10 ml of human embryonic stem cell medium containing DMEM/F-12, 20% knockout serum, 2 mM L-glutamine, 1% nonessential amino acids, 50 U/mL penicillin, 50 μg/mL streptomycin, 0.1 mM beta-mercaptoethanol. MEF-CM medium was collected and replaced with fresh medium every day, with collected medium filtered and either used immediately to propagate hESCs or stored at -20°C. 4ng/ml bFGF was added to MEF-CM before use for propagating hESCs. All reagents were obtained from Invitrogen (Carlsbad, CA) unless otherwise noted. This protocol was conducted under Virginia Commonwealth University- IACUC AM10103 approval.

### Propagation of human embryonic stem cells

Karyotypically normal diploid hESC (WA09, http://stemcells.nih.gov) were routinely passaged on MEFs in 35-mm dishes. A rapidly dividing, karyotypically aneuploid cell line BG01v (ATCC, VA) hESC was also routinely passaged on MEFs in 35-mm dishes, as described previously [2,9,10]. WA09 and BG01v hESCs were passaged as colonies by enzymatically methods every 3–4 days at subculturing ratios of 1:4 and maintained in hESC-medium. For use in our experiments, both WA09 and BG01v hESCs were transferred onto Matrigel^TM^ (BD Biosciences, CA) coated cell culture plates and propagated in MEF-CM.

### Generation of hESCs expressing CDK2AP1-specific shRNA and p53-specific shRNA

We have identified two potent shRNAs targeted to CDK2AP1 mRNA. Multiple shRNAs were obtained from commercially available sources (Open Biosystems, PA; Sigma-Aldrich, MO) and screened for their effectiveness. Control scrambled sequences were used similarly. To identify the shRNA clone that produced the strongest knockdown of CDK2AP1, hESCs were transduced with the different shRNA clones using lentiviral vectors and successfully transduced cells were selected by puromycin treatment (1 μg/ml). Following 6 days of selection, antibiotic-resistant colonies were harvested and RNA extracted. QPCR analyses using human CDK2AP1 specific primers were conducted. In our experiments, one shRNA (labeled as shRNA1 henceforth) (Open Biosystems, PA) produced the strongest knockdown and was used in subsequent experiments. For rescue experiments, validated CDK2AP1 shRNA (labeled as shRNA2 henceforth) (Sigma-Aldrich, MO) that target the 3’-UTR was used. In experiments involving analysis of the role of p53, expression was downregulated using lentiviral delivery of p53-specific shRNA (Addgene, MA, USA), followed by validation of knockdown by qPCR analyses.

### In vitro differentiation of hESCs and histopathology of hESC-derived embryoid bodies

To generate embryoid bodies (EBs), hESCs were dissociated using Trypsin and resuspended in growth medium devoid of bFGF. EB formation was facilitated using suspension culture by a hanging drop method as described previously [10,11]. In short, cells at a density of 25,000 cells/mL were suspended from a Petri dish lid in 20 μl droplets. After 2 days, the EBs were transferred to agarose plates at a density of 25–30 EBs/10 mL to facilitate further differentiation with media changes every 3–4 days, for a total differentiation duration of 18 days. The total number of viable EBs under each experimental condition was determined with each representative experiment performed in triplicate. Subsequently, EBs were prepared for histopathological analysis by fixation in 3.7% PFA in 1.5ml microfuge tubes at approximately 15–25 EBs per tube. Once fixed overnight, EBs were rinsed with PBS to remove PFA, resuspended in 200 μl melted 4% low melting point agarose (Sigma Aldrich, MO, USA) at 42°C and incubated for 2 hours to allow settling. Final pelleting and agarose solidification was performed with brief room temperature centrifugation at 500g. Agarose-embedded samples were processed for paraffin sectioning in a Leica TP1020 tissue processor. Hematoxylin and eosin (H&E) staining on microscope slide-mounted 5μm sections in a Leica Autostainer XL workstation was performed at the Dana-Farber/Harvard Cancer Center Rodent Histopathology Core Facility. Images were acquired using a Nikon TS-100 microscope using the default imaging parameters.

### RNA isolation, real-time reverse transcription polymerase chain reaction, and gene expression analysis

RNA was isolated from hESCs and EBs under different conditions using RNeasy kit (Qiagen, CA, USA), according to the manufacturer’s protocols and quantified using BioMate3 UV-VIS Spectrophotometer (Thermo Scientific, MA, USA). cDNA was synthesized from 1 μg of RNA using cDNA reverse transcription kit (Applied Biosystems, CA). Gene expression within different samples was analyzed using quantitative real time RT-PCR (QPCR). QPCR was performed in an ABI HT7900 system (Applied Biosystems, CA) and the data were acquired using sequence detection system software (SDS v2.2.1, Applied Biosystems, CA). Gene expression data (three replicates) were acquired and SDS software was used to estimate differential gene expression using ΔCT quantification methods. Endogenous GAPDH was used for normalization. Commercially available primers for CDK2AP1 and OCT4 were obtained from Origene, MD, while other primers summarized in S1 Table were obtained from Integrated DNA Technologies, IA.

### Antibodies and immunocytochemical analysis

Under different experimental conditions, hESCs were seeded onto four chambered glass slides. Paraformaldehyde (PFA, 4%) in PBS was used for fixation, permeabilization for intracellular markers was achieved with 0.2% Triton X-100 in PBS and blocked with normal goat serum. Fixed cells were incubated with primary antibodies: CDK2AP1 (Santa Cruz, CA, USA) and Anti-Phospho-Histone-3 (Cell Signaling, MA, USA). Goat anti-rabbit IgG conjugated to Alexa 594 (Invitrogen, CA,) was used as a secondary antibody. Fluorescent images were acquired using a Cool- Snap EZ camera (Photometrics, Tucson, AZ) mounted on a Nikon Eclipse TE 2000-S inverted microscope (Nikon, Melville, NY) with attached image analysis software. All image settings were controlled for uniform acquisition between samples. Specifically, uniform exposure time was maintained for images acquired from experimental samples as well as negative controls for background subtraction. For experiments involving determination of mitotic index based on phospho-histone 3 staining, random fields were selected and counting was performed in triplicate by counting around 500 cells during each analyses. Mitotic index was calculated by dividing the number of phospho-histone 3 positive cells, over the total number of cells.

### Western blot analysis

Western Blot was carried out as previously described [12]. Cells for analysis were harvested by trypsinization, centrifuged at 1000 rpm and washed once with ice-cold PBS buffer. Cells were then lysed using the Total Protein Extraction Kit (EMD Millipore, MA)) based on protocol provided by the manufacturer. Cell lysates were then subjected to Western blot analyses using specific antibodies to various cyclins. Cell lysates were prepared from wild type and CDK2AP1 knockdown WA09 hESCs and analyzed for CDK2AP1, OCT4, NANOG, p53, Cyclin A1 expression by Western blot using specific antibodies. The CDK2AP1, OCT4, NANOG and Cyclin A1 antibodies were obtained from Santa Cruz, CA, USA, while p53 antibody was obtained from Cell Signaling, MA, USA. Details of antibodies and sources are provided in S2 Table. Appropriate infrared emitting-conjugated secondary antibodies were obtained from Invitrogen, CA. Detection was then carried out using the Odyssey Infrared Imaging System (Li-Cor Biosciences, NE). The quantitative analysis of the Western Blot bands was carried out using the ImageJ software [13].

### Cell cycle profile analysis

Cells to be analyzed were trypsinized, washed, stained with propidium iodide for 45 min at 37°C, filtered through a 30 μm mesh to eliminate clumps and subjected to cell cycle analysis on an Accuri™ C6 Flow Cytometer (BD Biosciences, CA). Data were analyzed using the software provided by the manufacturer and samples analyzed in triplicate.

## Results

### Knockdown of CDK2AP1 in hESCs reduces *OCT4* and *NANOG* expression

**Fig 1:**
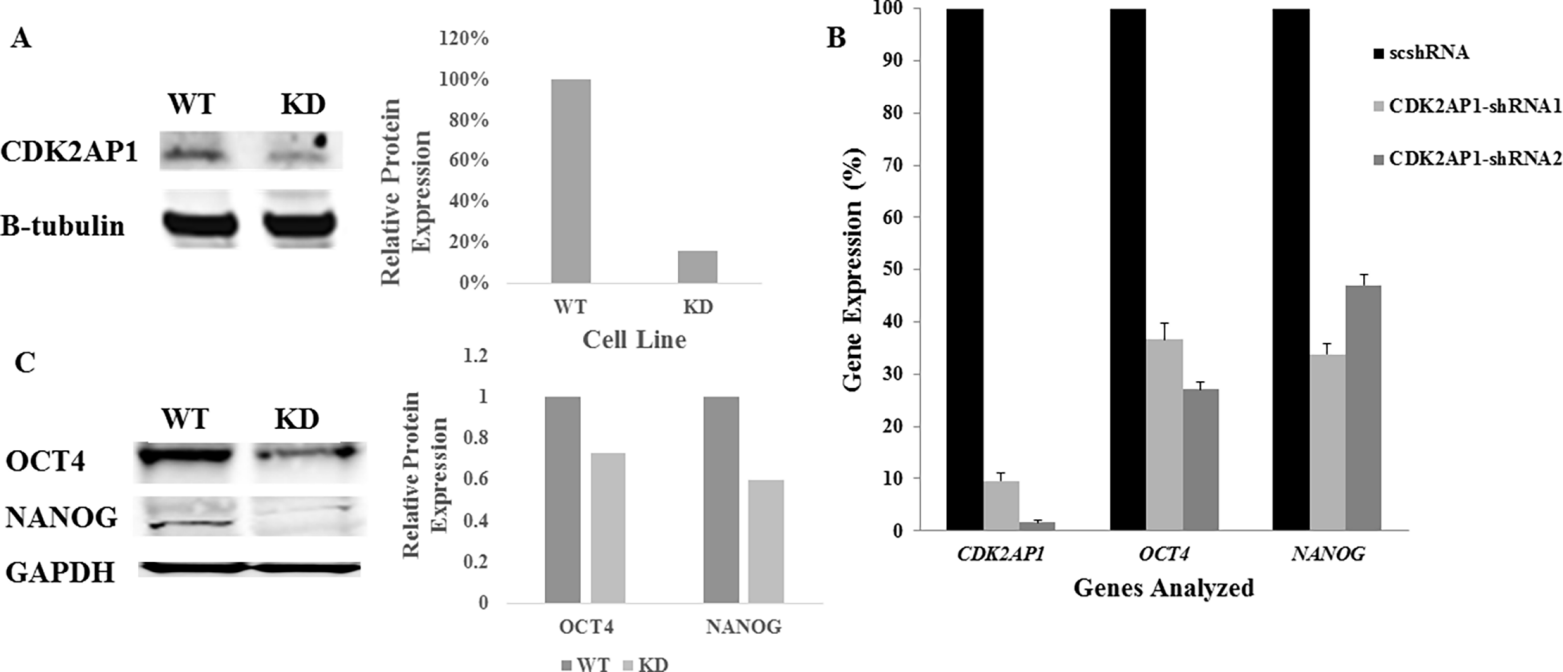
Knockdown of CDK2AP1 in hESCs reduces OCT4 and NANOG expression. A. Whole cell lysates were used to examine levels of CDK2AP1 protein in WA09 hESCs transduced with CDK2AP1 specific shRNA1 (right lane) or a control (left). Cells that were transduced with CDK2AP1 specific shRNA1 had lower CDK2AP1 protein levels. Densitometric analysis of the protein levels of the Western blot is presented in the right panel. B. WA09 hESCs that were transduced with CDK2AP1-shRNA1, CDK2AP1-shRNA2 or a scrambled shRNA (scshRNA) were examined for CDK2AP1 knockdown and for OCT4 and NANOG expression. Knockdown of CDK2AP1 using CDK2AP1-shRNA1 and CDK2AP1-shRNA2 resulted in reduction in OCT4 and NANOG expression when compared with hESCs that were transduced with the scshRNA(p-value < 0.05). Results are presented together with standard deviation from experiments conducted in triplicate. C. Whole cell lysates from WA09 hESCs transduced with CDK2AP1-shRNA1 (right lane) or scshRNA(left) were examined by Western Analysis and demonstrated reduction in OCT4 and NANOG levels. Densitometric analysis of the protein levels of the Western blot is presented in the right panel.

To investigate the effect of CDK2AP1 knockdown on the expression of the core pluripotent genes *OCT4* and *NANOG*, we used lentiviral delivery of shRNA1 to downregulate CDK2AP1 in WA09 hESC line. The CDK2AP1 shRNA clones tested utilize the U6 promoter to drive the expression of the shRNA’s, with the U6 promoter shown to be very active in undifferentiated hESCs [14]. Following the first passage after transduction, antibiotic-resistant colonies were selected upon exposure to puromycin at a concentration of 1μg/ml for 7 days. CDK2AP1 expression was then examined using Western blot (Fig 1A) and qPCR (Fig 1B). Based on qPCR analysis, we were able to achieve 90.5% knockdown of CDK2AP1, with statistical analysis indicating significant differences (p<0.05) in the expression patterns between hESCs transduced with the shRNA1 and the scrambled (sc) shRNA (Fig 1B). When knockdown was carried out using a different shRNA (shRNA2) 98% knockdown was achieved. Interestingly, we noted that the knockdown of CDK2AP1 using shRNA1 resulted in a statistically significant reduction (p <0.05) in *OCT4* and *NANOG* levels by 63.3% and 66.1% respectively (Fig 1B). In addition, when CDK2AP1 shRNA2 was used, it resulted in a reduction (p < 0.05) in *OCT4* and *NANOG* levels by 72.9% and 53% respectively. This data suggests a role for CDK2AP1 in affecting pluripotent gene expression of hESCs.

### Knockdown of CDK2AP1 in hESCs results in increased in vitro differentiation potential

**Fig 2:**
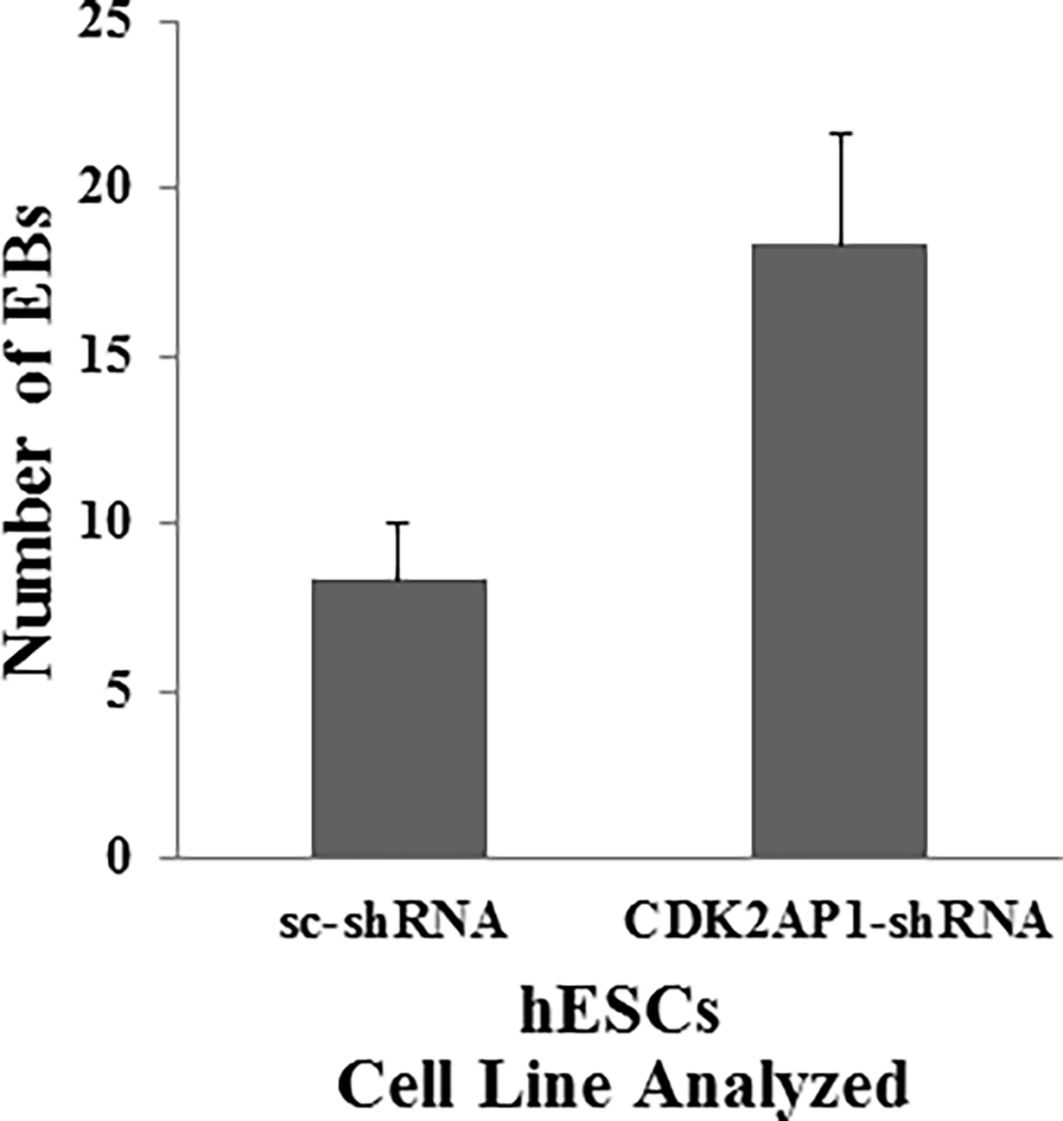
Knockdown of CDK2AP1 enhances differentiation. Knockdown of CDK2AP1 in hESC increased the number of generated EBs (p < 0.05). Results are presented together with standard deviation from experiments conducted in triplicate.

Previous studies that were performed on mESCs showed that *Cdk2ap1* knockout mESCs were resistant to differentiation and yielded lower number of embryoid bodies (EB) in which *Oct4* was still active [6,7]. These studies demonstrated the inability to silence *Oct4* expression in 8-day old EBs derived from *Cdk2ap1* knockout mESCs. We thus examined the effect of CDK2AP1 knockdown on EB formation and *Oct4* gene expression in these EBs derived from hESCs. Using the hanging drop method, we generated EBs from WA09 hESCs transduced with either a scrambled shRNA or CDK2AP1-shRNA1. Although a size difference was noted, with a nominal increase in the size of the knockdown- EBs, we did not observe statistically significant differences. However, our results indicate that a statistically significantly higher number of EBs (p <0.05) were generated from the knockdown when compared to wild-type cells, (Fig 2). Interestingly, this outcome suggests a potentially different role for CDK2AP1 in human pluripotent stem cells as the downregulation of this protein in mESCs resulted in a reduction in the number of EBs obtained. In addition, the knockdown of CDK2AP1 in human pluripotent stem cells enhanced their differentiation capability, yielding a significantly higher number of EBs which could be used in subsequent steps that could yield multiple terminally differentiated cell types.

A hallmark of differentiation in EBs is the formation of the three germ layers (ectoderm, mesoderm, endoderm). We next examined the expression of germ layer-specific markers in the EBs generated from CDK2AP1 knockdown WA09 hESCs by qPCR analysis. EBs generated from CDK2AP1 knockdown hESCs had a statistically significantly higher expression (p<0.05) of markers of all the three germ layers, when compared with EBs obtained from wild type hESCs (Table 1). This was specifically apparent in the expression of the mesoderm marker *T* (increased 27.6 fold) and in the ectoderm marker, *SOX1* (increased by 67 fold). We also conducted histopathological analysis of the EBs generated from CDK2AP1 knockdown WA09-hESCs and visually examined multiple EB sections. When compared with wild type EBs which consisted almost exclusively of neuroectoderm, EBs generated from CDK2AP1 knockdown WA09-hESCs exhibited evidence of ectoderm and mesoderm differentiation (S1 Fig). In addition, as is also widely acknowledged [11], we observed minimal evidence of endoderm formation based on histopathological analysis of EBs from both conditions. Taken together, these data demonstrated that the knockdown of CDK2AP1 enhanced the differentiation of hESCs and increased the expression of markers of all three germ layers in the derived EBs. To test whether CDK2AP1 is required for *OCT4* silencing in the generated EBs, we collected 8-day old EBs generated from sc-shRNA WA09 hESCs and CDK2AP1-shRNA WA09 hESCs and measured the levels of *OCT4* expression using qPCR analysis. Our results indicate that *OCT4* was equally silenced in both CDK2AP1 knockdown and wild type EBs (S2 Fig), thus indicating that CDK2AP1 is not required for *OCT4* silencing during the differentiation of hESCs.

**Table 1:** Knockdown of CDK2AP1 in WA09-hESCs results in EBs with enhanced expression of genes corresponding to the three germ layers. Quantitative PCR data showing the effect of CDK2AP1 knockdown on the expression of the germ layers markers in EBs derived from WA09 hESCs. EBs obtained from sc-shRNA transduced WA09s and EBs obtained from CDK2AP1-shRNA were harvested and the expression of markers of germ layers (Endoderm: *AFP* and *GATA4*. Mesoderm: *T* and *IGF2*. Ectoderm: *SOX1* and *NESTIN*) was examined by qPCR. Knockdown of CDK2AP1 significantly enhanced the expression of all markers of differentiation (p-value < 0.05).

Germ Layer Marker		Fold Change	p-value
**Endoderm**	*AFP*	1.9**↑**	0.01
	*GATA4*	2.1**↑**	2.0 E-04
**Mesoderm**	*T*	27.6**↑**	2.0 E-06
	*IGF2*	4.8**↑**	4.9 E-05
**Ectoderm**	*SOX1*	67.2**↑**	1.2 E-06
	*NESTIN*	3.5**↑**	0.002

### Knockdown of CDK2AP1 in hESCs reduces proliferation and alters cell cycle profile

Downregulation of *Cdk2ap1* in mESCs resulted in an increase in proliferation with hypersphosphorylation of pRb [8]. The differentiation capacity of these mESCs was extremely reduced and was only restored following the introduction of exogenous *Cdk2ap1*, or by expressing a mutated form of *pRb* that was resistant to phosphorylation [8]. This study presented a clear manifestation of the strong relationship between cell cycle regulation, pluripotency and differentiation. Being an inhibitor of CDK2, we initially expected that knockdown of CDK2AP1 would increase proliferation of hESCs and also reduce their differentiation capacity by pushing them more towards self-renewal. However, as mentioned above, we found that the knockdown of CDK2AP1 enhanced differentiation potential of the hESCs. We thus decided to examine the effect of the knockdown on the proliferation and cell cycle profile of hESCs.

**Fig 3:**
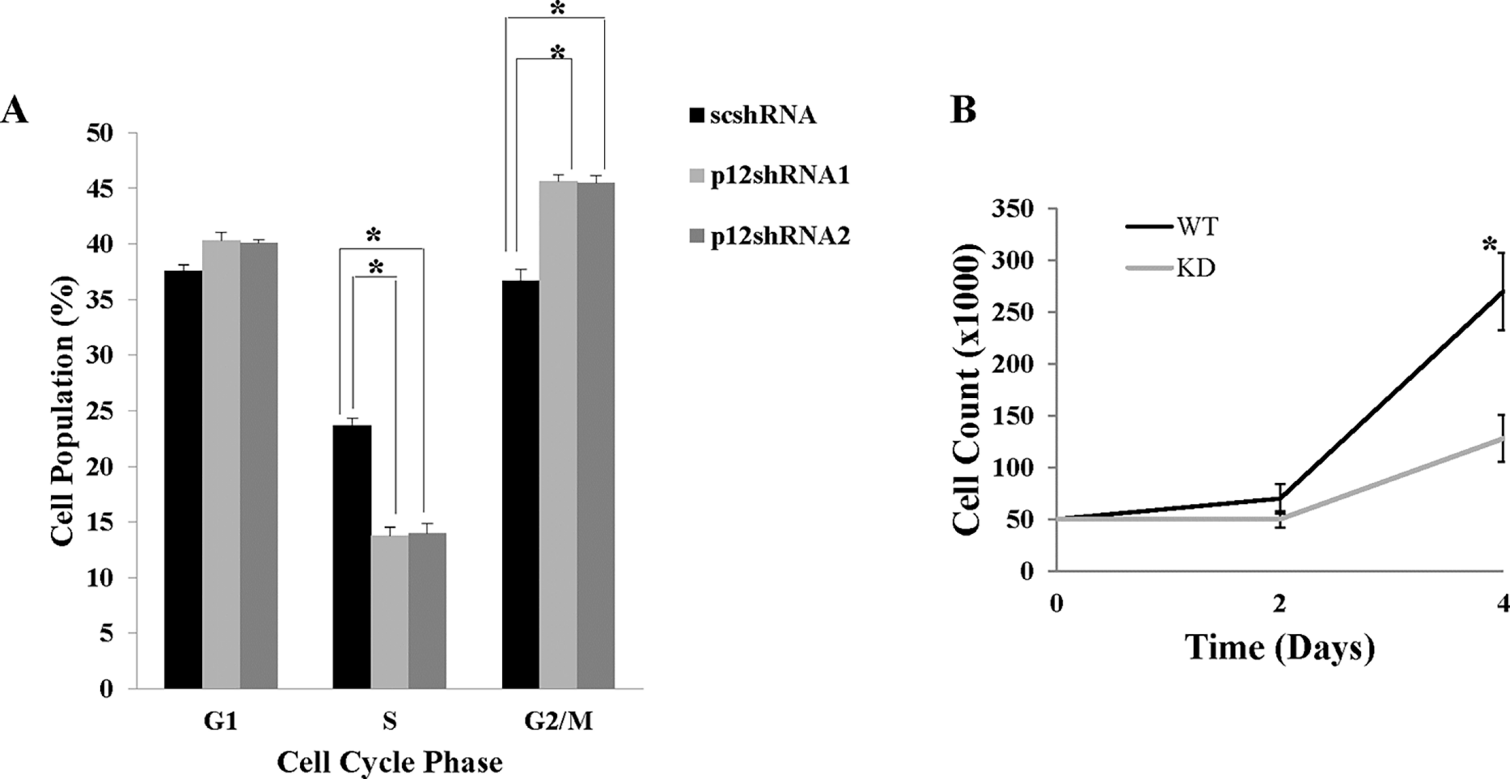
Knockdown of CDK2AP1 in hESC reduces proliferation and increases cells in the G2/M and decreases cells in the S phase of the cell cycle. A. Fifty thousand WA09 hESCs that were transduced with a scshRNA (WT) or CDK2AP1 shRNA1 (KD) were seeded per well in a 12-well plate. Cells were harvested and counted in triplicates at 2 and 4 days post seeding. (*- p-value < 0.05). Comparisons were made between WT and KD cells at respective time points). Results are presented together with standard deviation from experiments conducted in triplicate. B. CDK2AP1 wild type and knockdown WA09 hESCs were harvested and equal numbers were stained with propidium iodide (PI) and analyzed using an Accuri C6 flow cytometer. Results are presented together with standard deviation from experiments conducted in triplicate. (* = p < 0.05).

Fifty thousand WA09 hESCs that were transduced with either a scshRNA or with a CDK2AP1 specific shRNA1 were seeded per well in a 12-well plate and were counted at 2 and 4 days post seeding. We observed that the knockdown of CDK2AP1 significantly reduced the proliferation of hESCs (p<0.05) (Fig 3A). Cell cycle analysis demonstrated that the knockdown of CDK2AP1 significantly decreased cells in the S phase from 23% in wild type cells to an average of 14.6% in CDK2AP1 shRNA1 transduced cells (p-value = 0.0007). In addition, knockdown resulted in a significant increase in the percentage of cells in the G2/M (p-value = 0.0008) when compared with shRNA1 transduced cells from 36.7% to 45.6% (Fig 3B).

**Fig 4:**
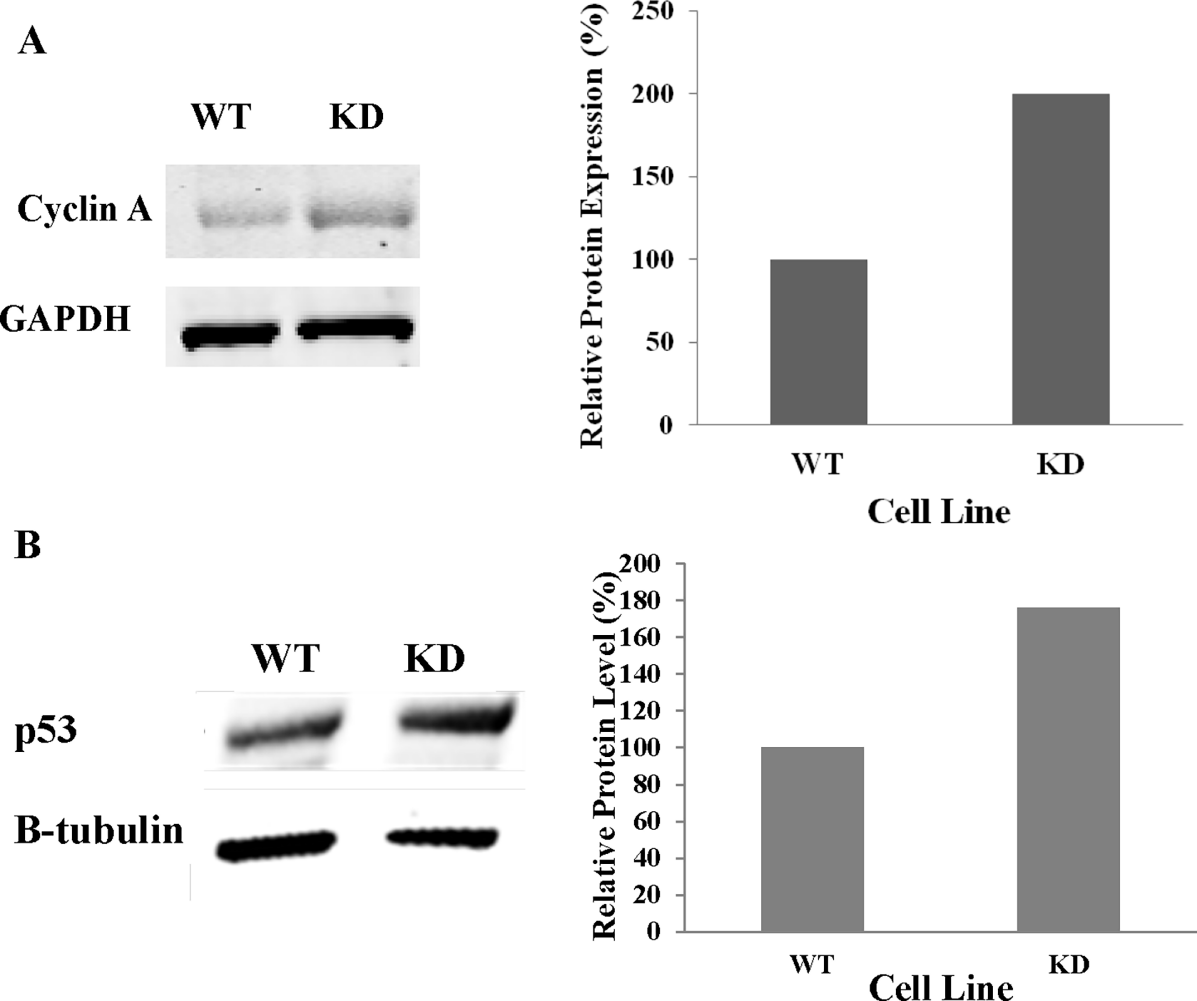
Knockdown of CDK2AP1 in hESCs increases Cyclin A and p53 protein levels. A. Western blot (left) shows an increase in Cyclin A levels in the CDK2AP1 knockdown hESCs when compared to wild type cells. Densitometric analysis of the protein levels of the Western blot is presented in the right panel. B. Whole cell lysates of wild type and CDK2AP1 knockdown WA09 hESCs were examined for p53 protein levels. We found that knockdown of CDK2AP1 increased p53 protein levels as shown by the western blot (left). Densitometric analysis of the protein level is presented in the right panel.

After we observed the significant increase in the percentage of CDK2AP1 knockdown hESCs that were in the G2/M phase of the cell cycle, we examined the protein levels of Cyclin A1 in those cells. It has been reported that increased Cyclin A1 can cause the accumulation of cells in the G2/M phase of the cell cycle, mitotic catastrophe and apoptosis [15]. Whole cell lysates of WA09 hESCs that were transduced with an empty vector or CDK2AP1 shRNA1 were examined for Cyclin A1 protein levels. We found that knockdown of CDK2AP1 increased the levels of Cyclin A1 by two fold (Fig 4A). We also examined the levels of phospho-Histone 3 (p-H3) which is detectable in cells that are in the late G2 and M phase of cell cycle [16]. Immunocytochemical analyses indicated that a significantly higher percentage (4.7%) of CDK2AP1knockdown WA09-hESCs stained positive for p-H3, while only (2.6%) of wild-type hESCs were positive (p<0.05) (S3 Fig).

These results indicate that CDK2AP1 may play a different role in hESCs when compared to mESCs. In *Cdk2ap1*-/- mESCs, differentiation capacity was compromised [5], whereas we found that CDK2AP1 knockdown WA09-hESCs had a significant reduction in the expression of the pluripotency genes, *OCT4* and *NANOG*, and generated higher number of EBs which had higher expression of candidate markers of the three germ layers when they were differentiated. *Cdk2ap1*-/- mESCs seemed to favor self-renewal over differentiation, which was apparent in their faster cell cycle and the hyperphosphorylation of the pRb. On the other hand, our results indicate that CDK2AP1 knockdown WA09-hESCs seem to favor differentiation over self-renewal, as evidenced by their reduced proliferation and reduced pluripotency gene expression.

### Knockdown of CDK2AP1 in hESCs results in an increase in p53 protein levels

After we observed the increase in Cyclin A1 protein levels following CDK2AP1 knockdown, we examined the levels of p53 in CDK2AP1 knockdown hESCs. It has been reported that increased p53 levels may increase Cyclin A1 leading to the accumulation of cells in the G2/M phase [15]. In addition, we suspected that following CDK2AP1 knockdown, its inhibition on CDK2 might be alleviated leading to upregulation of the oncogene E2F, which in turn could increase Cyclin A and p53 levels [17]. Whole cell lysate from CDK2AP1 wild type and knockdown WA09 hESCs were examined for p53 protein levels by Western blot analysis. We found that knockdown of CDK2AP1 resulted in a 1.76 fold increase in p53 protein levels (Fig 4B).

We next examined the expression of *p21*mRNA to test if it was affected by the increase in p53. Contrary to what was expected, qPCR analysis indicated that *p21* mRNA levels were reduced by ~60% in the CDK2AP1 knockdown WA09-hESC when compared with the wild type WA09-hESCs (S4 Fig). In studies assessing the role of microRNAs in hESCs, it has been reported that p53 can activate the micro-RNA’s: miR-302a, miR-302b, miR-302c and miR-302d, which can subsequently downregulate the *p21* mRNA when p53 is activated [18]. This study clearly explained the reason behind the non-functional p53-p21 axis of the G1/S phase check point in hESCs compared to somatic cells, which activate p21 in response to p53 stabilization and arrest in the G1 phase of the cell cycle. Although we observed a decrease in *p21* expression, we did not see an increase in G1 to S phase transition. It could be that CDK2AP1 knockdown have affected the levels of other G1 checkpoint regulators like p27 or p16.

### Simultaneous knockdown of CDK2AP1 and p53 prevents the reduction in *OCT4* and *NANOG* levels and also prevents the accumulation in the G2/M phase of the cell cycle

In a previous study, it was demonstrated that knockdown of p53 prevented retinoic acid mediated differentiation of hESCs [19]. The expression of pluripotency markers, *OCT4* and *NANOG* remained elevated even after 3 days of treatment with retinoic acid in the p53 knockdown hESCs. Further investigation revealed that upon the induction of differentiation in hESCs (by retinoic acid treatment), there was an enrichment in p53 binding to p53 responsive elements in the promoters of miR-34a and miR-145 [19]. MiR-34a is known to exacerbate p53 activation by inhibiting the p53-inhibitor, SIRT-1 [20], while miR-145 is known to suppress the expression of the pluripotency genes, *OCT4*, *SOX2*, and *KLF4* in hESCs and promote differentiation [21]. Taken together, we expect that the reduced expression of the pluripotency genes, *OCT4* and *NANOG* that is seen in the CDK2AP1 knockdown hESCs is due to p53 activation, which also affected the cell cycle profile and pluripotency.

**Fig 5:**
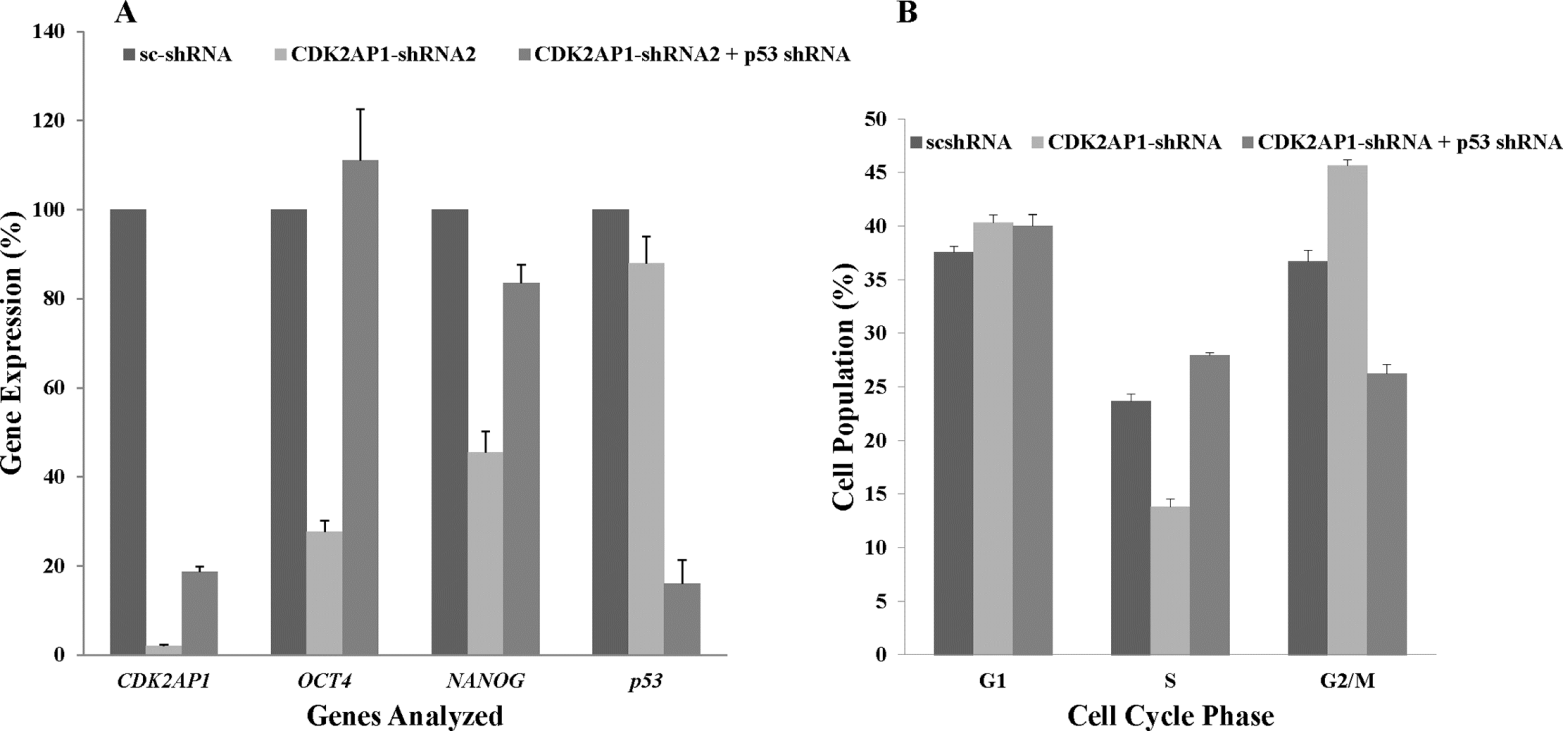
Simultaneous knockdown of CDK2AP1 and p53 prevents the reduction in *OCT4* and *NANOG* levels and the accumulation in the G2/M phase of the cell cycle. A. BG01v hESCs were transduced with scrambled shRNA (sc-shRNA), CDK2AP1 shRNA or co-transduced with CDK2AP1 and p53 shRNAs. The gene expression of CDK2AP1, OCT4, NANOG and p53 was analyzed in those cells by qPCR. We found that down regulating p53 with CDK2AP1 prevented the CDK2AP1-knockdown induced reduction in OCT4 and NANOG levels (p < 0.05). Results are presented together with standard deviation from experiments conducted in triplicate. B. When p53 is simultaneously downregulated with CDK2AP1, there was no reduction in cells in the S phase and no increase in cells in the G2/M phase of the cell cycle. Around 6% of the analyzed cells were in the sub G0/G1 phase of the cell cycle. Results are presented together with standard deviation from experiments conducted in triplicate. Cell cycle data of scshRNA and CDK2AP1-shRNA transduced hESCs from Fig 3B were included in this figure to facilitate comparison.

To investigate whether the observed phenotype seen in CDK2AP1 knockdown hESCs is p53 dependent, we co-transduced BG01v hESCs with p53 and CDK2AP1 shRNAs and assayed *OCT4* and *NANOG* expression by qPCR analysis. The double knockdown cells did not silence *OCT4* (p-value = 0.004) or *NANOG* (p-value = 0.0005) expression when compared with CDK2AP1 only knockdown cells (Fig 5A). Similarly, introduction of exogenous *CDK2AP1* prevented the reduction in *OCT4* and *NANOG* expression seen in CDK2AP1 knockdown hESCs (S5 Fig).

In experiments conducted with WA09-hESCs, we found that CDK2AP1 knockdown resulted in a decrease in the percentage of cells that were in the S phase and increased cells in the G2/M phase of the cell cycle (Fig 3B). To test if this shift in the cell cycle is p53 dependent, we examined the cell cycle of WA09 hESCs that were co-transduced with p53 and CDK2AP1 shRNAs. We found that these cells in which p53 and CDK2AP1 were downregulated did not accumulate in the G2/M phase and had a significantly higher percentage of cells in the S phase when compared to CDK2AP1 knockdown cells (Fig 5B). We noted a slight delay in S-G2/M transition in the double knockdown cells, which could be due to abnormalities in DNA synthesis as both CDK2AP1 and p53 regulate DNA replication [22,23].

## Discussion

The function of CDK2AP1 as initially assessed in mESCs indicated an important role in the differentiation and self-renewal of pluripotent stem cells [6,8]. Its deletion in mESCs resulted in an increase in self-renewal and a reduction in differentiation capacity, caused by a defect in the proper epigenetic silencing of *Oct4* during differentiation due to the delocalization of the Nucleosome Remodeling and Deacetylation (NuRD) complex from Oct4 promoter [6]. Therefore, it was concluded that Cdk2ap1 is required for epigenetic silencing of Oct4 during mESC differentiation. Other studies also demonstrated the significance of Cdk2ap1 as an important NuRD-associated factor and its role in the regulation of stemness and differentiation [24, 25]. Another study demonstrated that, *Cdk2ap1* -/- mESCs failed to differentiate due to increased phosphorylation of pRb which lead to persistent self-renewal [8]. Differentiation capacity was restored in those cells after introduction of an unphosphorylatable form of pRb or exogenous *Cdk2ap*.

To investigate the function of CDK2AP1 in human pluripotent stem cell self-renewal, pluripotency and differentiation potential, we knocked down CDK2AP1 expression in multiple human pluripotent stem cells by RNA interference. Using a lentiviral approach, we were able to achieve more than 98% knockdown. When we examined the levels of *OCT4* and *NANOG* in the CDK2AP1 knockdown hESCs, we found that the knockdown resulted in a decrease in the transcription of these genes (CDK2AP1-shRNA1 resulted in a 60% reduction in *OCT4* and *NANOG* and when CDK2AP1-shRNA2 was used, knockdown resulted in a 70% reduction in *OCT4* and 50% reduction in *NANOG* expression). These results indicated a possible difference in the role of CDK2AP1 in hESCs when compared to mESC, in which when *Cdk2ap1* was deleted, *Oct4* expression was sustained [6]. Indeed, in mESC knockout model, Cdk2ap1 is completely obliterated whereas in hESCs, some CDK2AP1 expression remains. Additionally, following lentiviral delivery of shRNA and antibiotic selection, the resulting cells are heterogeneous with varying numbers of shRNA integrations. Therefore, cells with fewer integrations may display antibiotic resistance, yet retain relatively higher levels of CDK2AP1 that are sufficient to change the phenotype. We acknowledge that gene-editing techniques would be optimal and hope to be able to conduct future studies to generate null alleles of hESC lines that are uniform. However, it is also useful to note that the observed phenotype with hESCs is similar with multiple shRNAs (with completely different sequences and towards different regions of the CDK2AP1 transcript) in two different hESC lines, with the odds of off-target effects being very limited. Given the fact that hESCs are representative of cells isolated from post-implantation embryos, this could point to some of the observed differences between the mouse knockout and our knockdown studies in hESCs, and also alludes to a different role for CDK2AP1 in pre- and post-implantation embryos.

Our results also indicated that CDK2AP1 knockdown hESCs had a greater propensity to differentiate when EB formation was induced. In parallel experiments, we also obtained results that indicated a statistically significantly higher number of EBs (p <0.05) were generated from the knockdown when compared to wild-type hiPSCs (data not shown), leading us to infer the similarities in outcomes across multiple pluripotent stem cell types. Our future studies could thus focus on a broader assessment of similarities and differences in CDK2AP1 expression and knockdown effects in hESC and hiPSCs. When we examined the expression of markers of the germ layers in 18–21 day old EBs, we found that EBs generated from CDK2AP1 knockdown hESCs exhibited higher expression of candidate markers of all the three germ layers. The difference was especially evident in the expression of mesoderm (27.6 fold increase in *T*) and in the ectoderm markers (67.2 fold increase in *SOX1*). Histopathological analysis also revealed that EBs generated from CDK2AP1 knockdown hESCs primarily demonstrated mesoderm and ectoderm differentiation, while EBs generated from wild type hESCs routinely demonstrated exclusive differentiation towards neuroectoderm. Taken together, our results indicate that knockdown of CDK2AP1 in hESCs enhances differentiation by yielding more EBs that have significantly higher expression of markers of all three germ layers.

In vitro studies have revealed that CDK2AP1 mainly functions by regulating the S phase of the cell cycle, with studies showing that binding of CDK2AP1 to the DNA polymerase alpha/primase complex specifically prevents the initiation step of the DNA replication process [22]. It has also been shown to regulate G1/S phase progression by inhibiting CDK2 and targeting it for proteolysis [26]. Given the close connection between cell cycle and pluripotency, we next examined the effect of CDK2AP1 knockdown on the proliferation and the cell cycle of hESCs [27]. We initially observed that CDK2AP1 knockdown hESCs were proliferating significantly slower and had to be sub-cultured less frequently. When the cell cycle profile of these cells was examined by propidium iodide staining and flow cytometry analysis, we found that these cells accumulated in the G2/M phase of the cell cycle and had a significantly lower percentage of cells in the S phase of the cell cycle. We also found that a higher percentage of these cells stained positive for p-H3, which is detected in cells that are in the late G2 and M phase of the cell cycle. In addition, Cyclin A1 and p53 protein levels were increased following CDK2AP1 knockdown by 2 and 1.76 folds respectively. It has been reported previously, that increased p53 levels can increase Cyclin A1which in turn can cause G2/M arrest [15].

Increased p53 levels can also cause arrest in the G2/M phase of the cell cycle in a number of ways that are independent of Cyclin A1. It is known that when levels of p53 are increased, it results in the induction of the expression of the 14-3-3σ, a gene originally discovered through its expression in differentiating epithelial cells and a member of the 14-3-3 protein family [28,29]. Through a p53-responsive element located 1.8 kb upstream of its transcription start site, 14-3-3σ is induced upon p53 binding and inhibits G2/M progression by sequestering CDK1 in the cytoplasm [28]. In addition, p53 can exert its inhibition of the G2/M transition by decreasing intracellular levels of cyclin B1 protein and attenuating the activity of the cyclin B1 promoter [30]. Furthermore, p53 has also been shown to repress the promoter of CDK1, which is another way through which G2/M transition is regulated by p53 [31]. We observed that when p53 and CDK2AP1 were both downregulated, there was minimal reduction in OCT4 and NANOG expression, suggesting the observed reduction in their levels following CDK2AP1 may be p53 dependent. A recent study showed that upon the induction of differentiation of hESCs, there was an enrichment in p53 binding to p53 responsive elements in the promoters of miR-145 [19], which then suppresses the expression of the pluripotency genes, OCT4, SOX2 and KLF4 in hESCs and promotes differentiation [21]. In addition, when the cell cycle profile of WA09 hESCs that were co-transduced with CDK2AP1 and p53 shRNAs was examined, we found that the introduction of the p53 shRNA prevented the decrease in S phase and the increase in the G2/M phase of the cell cycle that was seen in the CDK2AP1 knockdown only hESCs. This data suggests that the change in the cell cycle profile following CDK2AP1 knockdown may be caused due to an increase in p53 levels. Being an inhibitor of CDK2 we expect that the knockdown of CDK2AP1 might have temporarily alleviated this inhibition leading to faster progression into S phase and an aberrant upregulation of the oncogene E2F. This in turn may cause a p16 (ARF) mediated degradation of the p53 inhibitor MDM2 leading to an increase in p53 levels (Fig 4B) [32].

Although CDK2AP1knockdown in hESCs resulted in a decrease in OCT4 and NANOG levels, these cells differentiated better than wild types. The knockdown of CDK2AP1 improved the generation of EBs and enhanced the expression of all tested markers of the three germ layers. Overall, our results indicate that hESCs exhibit reduced self-renewal potential following CDK2AP1 knockdown, a significantly lower percentage of cells in the S phase, and higher percentage were in the G2/M phase when compared to wild type cells. The observed effect upon knockdown of CDK2AP1 in hESCs is most likely a result of its role in cell cycle control. However, it is important to note that cell cycle regulation and pluripotency are highly interconnected and a change in one will most likely affect the other. Overall, our results lead us to the conclusion that the knockdown of CDK2AP1 in hESCs lowers the threshold of differentiation by decreasing self-renewal and increasing the susceptibility of these cells to differentiate.

## Supporting information

S1 Fig Knockdown of CDK2AP1 enhances differentiation. Shown are images of hematoxylin and eosin-stained histopathologic sections of EBs generated from CDK2AP1 knockdown WA09 hESCs. Representative ectodermal (neuroepithelial) and mesodermal (fibrous connective) is exhibited. Scale bar represents 100 μm.
(TIF)

S2 Fig CDK2AP1 is not required for OCT4 silencing during the differentiation of hESCs. To investigate if CDK2AP1 is required for proper silencing of OCT4 during hESC, 8-day old EBs generated from CDK2AP1 wild type (sc-shRNA) and knockdown (CDK2AP1-shRNA) hESCs were harvested and the levels of CDK2AP1 and OCT4 measured by qPCR. Results indicate that CDK2AP1 knockdown hESCs were able to shut down OCT4 expression to the same levels seen in the wild type EBs (p-value = 0.88). Results are presented together with standard deviation from experiments conducted in triplicate.
(TIF)

S3 Fig Knockdown of CDK2AP1 in WA09 hESC increases the level of phospho-histone 3. WA09 hESCs were transduced with a scrambled shRNA (sc-shRNA) or with CDK2AP1- shRNA1. Cells were fixed and stained using a phospho-Histone 3 specific antibody. Around 500 cells were counted in randomly selected fields and the percentage of p-H3 positive cells was calculated. A. Shows that the percentage of p-H3 positive cells. Results are presented together with standard deviation from experiments conducted in triplicate. B. Shows the p-H3 staining, DAPI, β-Tubulin and a merge picture in both sc-shRNA and CDK2AP1-shRNA transduced cells. Scale bar represents 50 μm.
(TIF)

S4 Fig Knockdown of CDK2AP1 in hESCs reduces p21 expression. Quantitative PCR analysis showing the levels of *CDK2AP1* and *p21* expression in wild type and CDK2AP1 knockdown WA09 hESCs. Knockdown of CDK2AP1 resulted in a 63% reduction in *p21* expression (p < 0.05. Comparisons were made between sc-shRNA and CDK2AP1-shRNA1 transduced cells for each gene analyzed). Results are presented together with standard deviation from experiments conducted in triplicate.
(TIF)

S5 Fig Introduction of exogenous *CDK2AP1* simultaneously with CDK2AP1 shRNA2 prevents reduction in *OCT4* and *NANOG* expression. BG01v hESCs were transduced with sc-shRNA or with exogenous *CDK2AP1* + CDK2AP1 shRNA2 and analyzed by qPCR for *OCT4* and *NANOG* expression. Prevention of knockdown by introducing exogenous *CDK2AP1* prevents the reduction in *OCT4* and *NANOG* expression seen in CDK2AP1 knockdown hESCs (p> 0.05. Comparisons were made between sc-shRNA and CDK2AP1-shRNA2 + *CDK2AP1* for each gene analyzed). Results are presented together with standard deviation from experiments conducted in triplicate.
(TIF)

S1 Table Sequences of primers used in qPCR analysis. (DOCX)

S2 Table List of antibodies and sources used in immunocytochemical and Western blot analysis. (DOCX)

## Abbreviations

HESC: Human Embryonic Stem Cell
CDK2: Cyclin Dependent Kinase 2
CDK2AP1: Cyclin Dependent Kinase-2 Associated Protein-1
ARF: Alternative Reading Frame
shRNA: Short Hairpin RNA
sc-shRNA: Scrambled shRNA
BrdU: Bromodeoxyuridine
X-gal: Betagalactosidase
ICC: Immunocytochemistry

## References

[1] Ramalho-Santos M, Yoon S, Matsuzaki Y, Mulligan RC, Melton DA. "Stemness": transcriptional profiling of embryonic and adult stem cells. Science. 2002;298(5593):597–600. doi: 10.1126/science.1072530 12228720 10.1126/science.1072530

[2] Rao RR, Calhoun JD, Qin X, Rekaya R, Clark JK, Stice SL. Comparative transcriptional profiling of two human embryonic stem cell lines. Biotechnol Bioeng. 2004;88(3):273–86. doi: 10.1002/bit.20245 15493035 10.1002/bit.20245

[3] Rao RR, Stice SL. Gene expression profiling of embryonic stem cells leads to greater understanding of pluripotency and early developmental events. Biol Reprod. 2004;71(6):1772–8. doi: 10.1095/biolreprod.104.030395 15140800 10.1095/biolreprod.104.030395

[4] Sharov AA, Piao Y, Matoba R, Dudekula DB, Qian Y, VanBuren V, et al Transcriptome analysis of mouse stem cells and early embryos. PLoS Biol. 2003;1(3):E74 doi: 10.1371/journal.pbio.0000074 14691545 10.1371/journal.pbio.0000074 PMC300684

[5] Kim Y, McBride J, Kimlin L, Pae EK, Deshpande A, Wong DT. Targeted inactivation of p12, CDK2 associating protein 1, leads to early embryonic lethality. PLoS One. 2009;4(2):e4518 doi: 10.1371/journal.pone.0004518 19229340 10.1371/journal.pone.0004518 PMC2641017

[6] Deshpande AM, Dai YS, Kim Y, Kim J, Kimlin L, Gao K, et al Cdk2ap1 is required for epigenetic silencing of Oct4 during murine embryonic stem cell differentiation. J Biol Chem. 2009;284(10):6043–7. doi: 10.1074/jbc.C800158200 19117947 10.1074/jbc.C800158200 PMC2649091

[7] Deshpande AM, Khalid O, Kim JJ, Kim Y, Lindgren A, Clark AT, et al Cdk2ap2 is a novel regulator for self-renewal of murine embryonic stem cells. Stem Cells Dev. 2012;21(16):3010–8. doi: 10.1089/scd.2012.0007 22548356 10.1089/scd.2012.0007 PMC3475145

[8] Kim Y, Deshpande A, Dai Y, Kim JJ, Lindgren A, Conway A, et al Cyclin-dependent kinase 2-associating protein 1 commits murine embryonic stem cell differentiation through retinoblastoma protein regulation. J Biol Chem. 2009;284(35):23405–14. doi: 10.1074/jbc.M109.026088 19564334 10.1074/jbc.M109.026088 PMC2749114

[9] Abraham S, Sheridan SD, Laurent LC, Albert K, Stubban C, Ulitsky I, et al Propagation of human embryonic and induced pluripotent stem cells in an indirect co-culture system. Biochem Biophys Res Commun. 2010;393(2):211–6. doi: 10.1016/j.bbrc.2010.01.101 20117095 10.1016/j.bbrc.2010.01.101 PMC2834855

[10] Abraham S, Sheridan SD, Miller B, Rao RR. Stable propagation of human embryonic and induced pluripotent stem cells on decellularized human substrates. Biotechnol Prog. 2010;26(4):1126–34. doi: 10.1002/btpr.412 20730767 10.1002/btpr.412

[11] Sheridan SD, Surampudi V, Rao RR. Analysis of embryoid bodies derived from human induced pluripotent stem cells as a means to assess pluripotency. Stem Cells Int. 2012;2012:738910 doi: 10.1155/2012/738910 22550517 10.1155/2012/738910 PMC3328185

[12] Adams BR, Golding SE, Rao RR, Valerie K. Dynamic dependence on ATR and ATM for double-strand break repair in human embryonic stem cells and neural descendants. PLoS One. 2010;5(4):e10001 doi: 10.1371/journal.pone.0010001 20368801 10.1371/journal.pone.0010001 PMC2848855

[13] Schneider CA, Rasband WS, Eliceiri KW. NIH Image to ImageJ: 25 years of image analysis. Nat Methods. 2012;9(7):671–5. 22930834 10.1038/nmeth.2089 PMC5554542

[14] Xia X, Zhang SC. Genetic modification of human embryonic stem cells. Biotechnol Genet Eng Rev. 2007;24:297–309. 18059639 10.1080/02648725.2007.10648105 PMC2950622

[15] Rivera A, Mavila A, Bayless KJ, Davis GE, Maxwell SA. Cyclin A1 is a p53-induced gene that mediates apoptosis, G2/M arrest, and mitotic catastrophe in renal, ovarian, and lung carcinoma cells. Cell Mol Life Sci. 2006;63(12):1425–39. doi: 10.1007/s00018-006-5521-5 16799873 10.1007/s00018-006-5521-5 PMC11136098

[16] Van Hooser A, Goodrich DW, Allis CD, Brinkley BR, Mancini MA. Histone H3 phosphorylation is required for the initiation, but not maintenance, of mammalian chromosome condensation. J Cell Sci. 1998;111 (Pt 23):3497–506. 9811564 10.1242/jcs.111.23.3497

[17] Soucek T, Pusch O, Hengstschlager-Ottnad E, Adams PD, Hengstschlager M. Deregulated expression of E2F-1 induces cyclin A- and E-associated kinase activities independently from cell cycle position. Oncogene. 1997;14(19):2251–7. doi: 10.1038/sj.onc.1201061 9178900 10.1038/sj.onc.1201061

[18] Dolezalova D, Mraz M, Barta T, Plevova K, Vinarsky V, Holubcova Z, et al MicroRNAs regulate p21(Waf1/Cip1) protein expression and the DNA damage response in human embryonic stem cells. Stem Cells. 2012;30(7):1362–72. doi: 10.1002/stem.1108 22511267 10.1002/stem.1108

[19] Jain AK, Allton K, Iacovino M, Mahen E, Milczarek RJ, Zwaka TP, et al p53 regulates cell cycle and microRNAs to promote differentiation of human embryonic stem cells. PLoS Biol. 2012;10(2):e1001268 doi: 10.1371/journal.pbio.1001268 22389628 10.1371/journal.pbio.1001268 PMC3289600

[20] Yamakuchi M, Ferlito M, Lowenstein CJ. miR-34a repression of SIRT1 regulates apoptosis. Proc Natl Acad Sci U S A. 2008;105(36):13421–6. doi: 10.1073/pnas.0801613105 18755897 10.1073/pnas.0801613105 PMC2533205

[21] Xu N, Papagiannakopoulos T, Pan G, Thomson JA, Kosik KS. MicroRNA-145 regulates OCT4, SOX2, and KLF4 and represses pluripotency in human embryonic stem cells. Cell. 2009;137(4):647–58. doi: 10.1016/j.cell.2009.02.038 19409607 10.1016/j.cell.2009.02.038

[22] Matsuo K, Shintani S, Tsuji T, Nagata E, Lerman M, McBride J, et al p12(DOC-1), a growth suppressor, associates with DNA polymerase alpha/primase. FASEB J. 2000;14(10):1318–24. 10877824 10.1096/fj.14.10.1318

[23] Zhou J, Prives C. Replication of damaged DNA in vitro is blocked by p53. Nucleic Acids Res. 2003;31(14):3881–92. 12853603 10.1093/nar/gkg468 PMC165982

[24] Bode D, Yu L, Tate P, Pardo M, Choudhary J. Characterization of Two Distinct Nucleosome Remodeling and Deacetylase (NuRD) Complex Assemblies in Embryonic Stem Cells. Mol Cell Proteomics. 2016;15(3):878–91. doi: 10.1074/mcp.M115.053207 26714524 10.1074/mcp.M115.053207 PMC4813707

[25] Kim JJ, Khalid O, Vo S, Sun HH, Wong DT, Kim Y. A novel regulatory factor recruits the nucleosome remodeling complex to wingless integrated (Wnt) signaling gene promoters in mouse embryonic stem cells. J Biol Chem. 2012;287(49):41103–17. doi: 10.1074/jbc.M112.416545 23074223 10.1074/jbc.M112.416545 PMC3510811

[26] Shintani S, Ohyama H, Zhang X, McBride J, Matsuo K, Tsuji T, et al p12(DOC-1) is a novel cyclin-dependent kinase 2-associated protein. Mol Cell Biol. 2000;20(17):6300–7. 10938106 10.1128/mcb.20.17.6300-6307.2000 PMC86104

[27] Wong DT, Kim JJ, Khalid O, Sun HH, Kim Y. Double edge: CDK2AP1 in cell-cycle regulation and epigenetic regulation. J Dent Res. 2012;91(3):235–41. doi: 10.1177/0022034511420723 21865592 10.1177/0022034511420723 PMC3275332

[28] Hermeking H, Lengauer C, Polyak K, He TC, Zhang L, Thiagalingam S, et al 14-3-3sigma is a p53-regulated inhibitor of G2/M progression. Mol Cell. 1997;1(1):3–11. 9659898 10.1016/s1097-2765(00)80002-7

[29] Prasad GL, Valverius EM, McDuffie E, Cooper HL. Complementary DNA cloning of a novel epithelial cell marker protein, HME1, that may be down-regulated in neoplastic mammary cells. Cell Growth Differ. 1992;3(8):507–13. 1390337

[30] Innocente SA, Abrahamson JL, Cogswell JP, Lee JM. p53 regulates a G2 checkpoint through cyclin B1. Proc Natl Acad Sci U S A. 1999;96(5):2147–52. 10051609 10.1073/pnas.96.5.2147 PMC26751

[31] Passalaris TM, Benanti JA, Gewin L, Kiyono T, Galloway DA. The G(2) checkpoint is maintained by redundant pathways. Mol Cell Biol. 1999;19(9):5872–81. 10454534 10.1128/mcb.19.9.5872 PMC84436

[32] Zhang Y, Xiong Y, Yarbrough WG. ARF promotes MDM2 degradation and stabilizes p53: ARF-INK4a locus deletion impairs both the Rb and p53 tumor suppression pathways. Cell. 1998;92(6):725–34. 9529249 10.1016/s0092-8674(00)81401-4

