# Supplementary figures and images for: Knockdown of CDK2AP1 in human embryonic stem cells reduces the threshold of differentiation
Source: PLoS One. 2018 May 7;13(5):e0196817. doi: 10.1371/journal.pone.0196817 (PMC5937771; doi:10.1371/journal.pone.0196817)

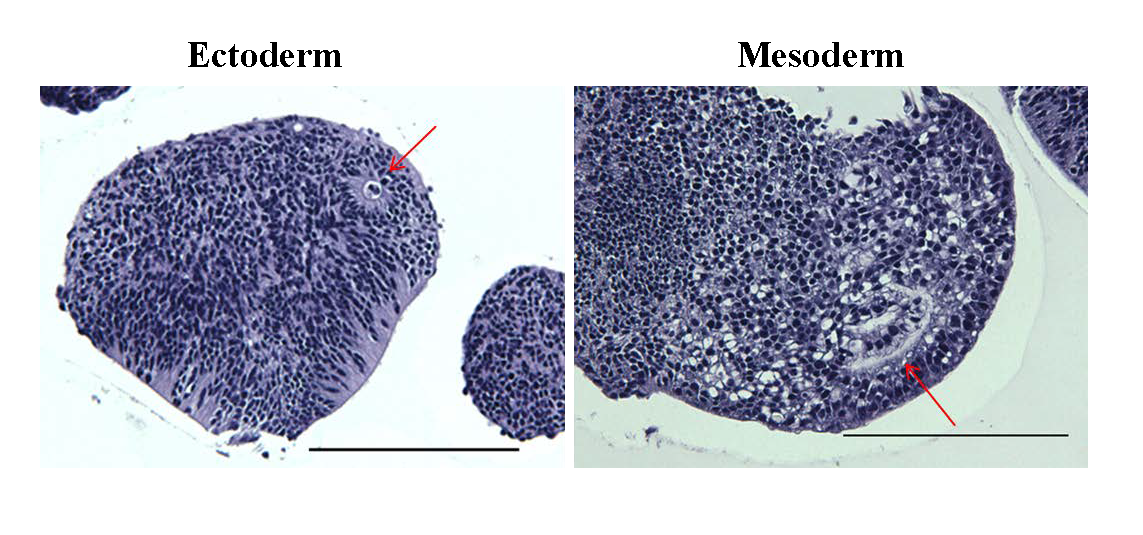

Supplement: S1 Fig — Shown are images of hematoxylin and eosin-stained histopathologic sections of EBs generated from CDK2AP1 knockdown WA09 hESCs. Representative ectodermal (neuroepithelial) and mesodermal (fibrous connective) is exhibited. Scale bar represents 100 μm. (TIF) [file pone.0196817.s001.tif]

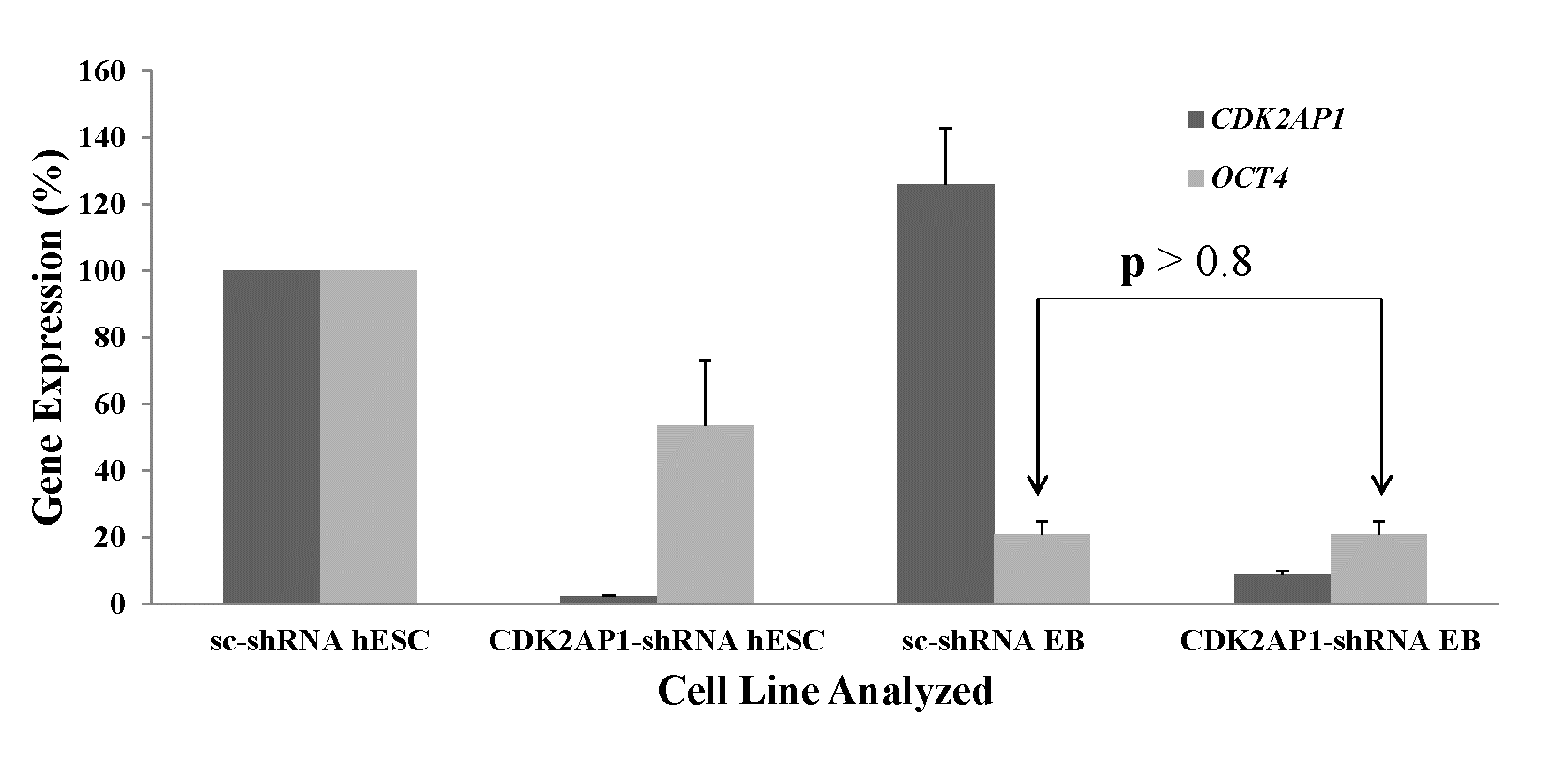

Supplement: S2 Fig — To investigate if CDK2AP1 is required for proper silencing of OCT4 during hESC, 8-day old EBs generated from CDK2AP1 wild type (sc-shRNA) and knockdown (CDK2AP1-shRNA) hESCs were harvested and the levels of CDK2AP1 and OCT4 measured by qPCR. Results indicate that CDK2AP1 knockdown hESCs were able to shut down OCT4 expression to the same levels seen in the wild type EBs (p-value = 0.88). Results are presented together with standard deviation from experiments conducted in triplicate. (TIF) [file pone.0196817.s002.tif]

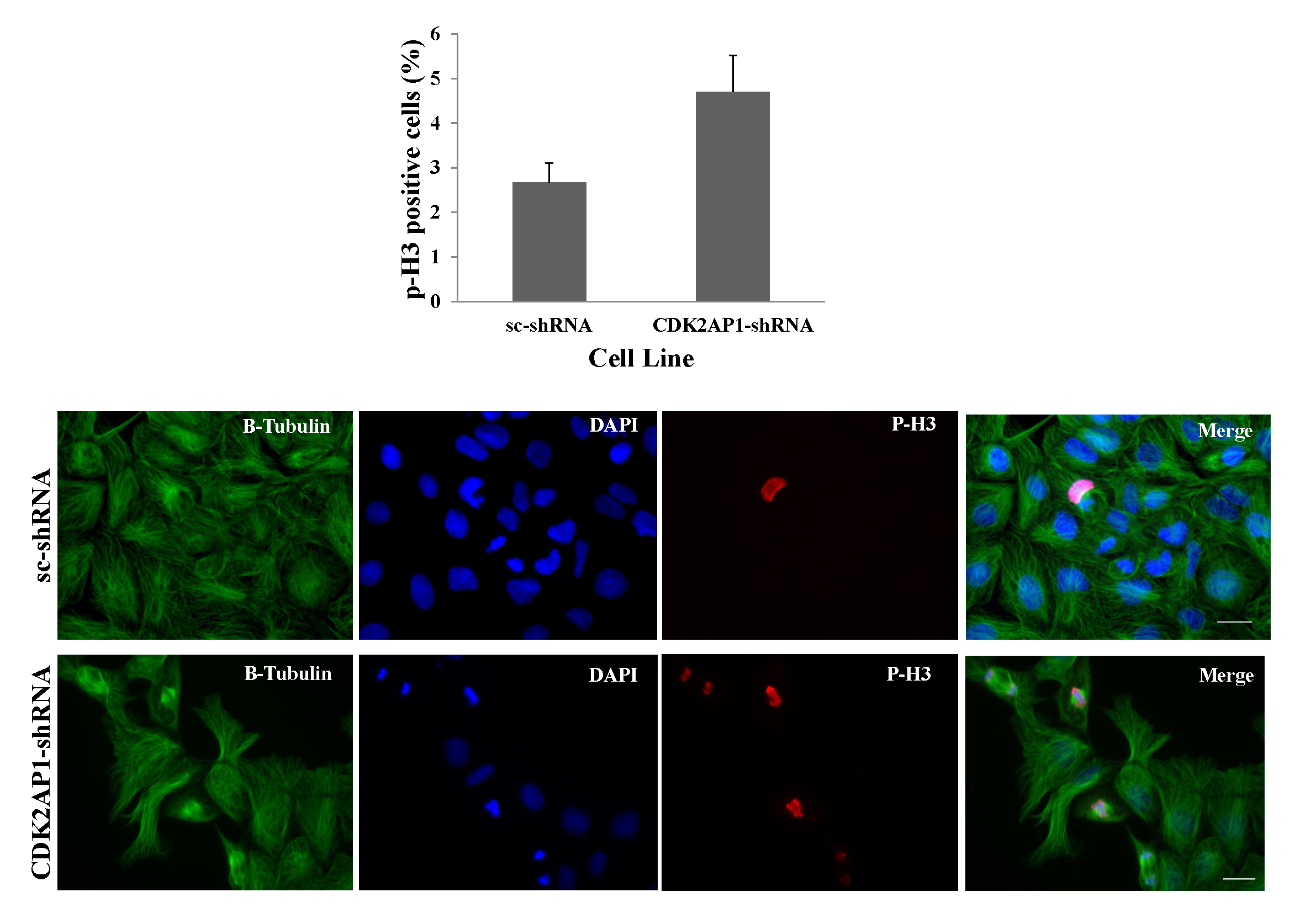

Supplement: S3 Fig — WA09 hESCs were transduced with a scrambled shRNA (sc-shRNA) or with CDK2AP1- shRNA1. Cells were fixed and stained using a phospho-Histone 3 specific antibody. Around 500 cells were counted in randomly selected fields and the percentage of p-H3 positive cells was calculated. A. Shows that the percentage of p-H3 positive cells. Results are presented together with standard deviation from experiments conducted in triplicate. B. Shows the p-H3 staining, DAPI, β-Tubulin and a merge picture in both sc-shRNA and CDK2AP1-shRNA transduced cells. Scale bar represents 50 μm. (TIF) [file pone.0196817.s003.tif]

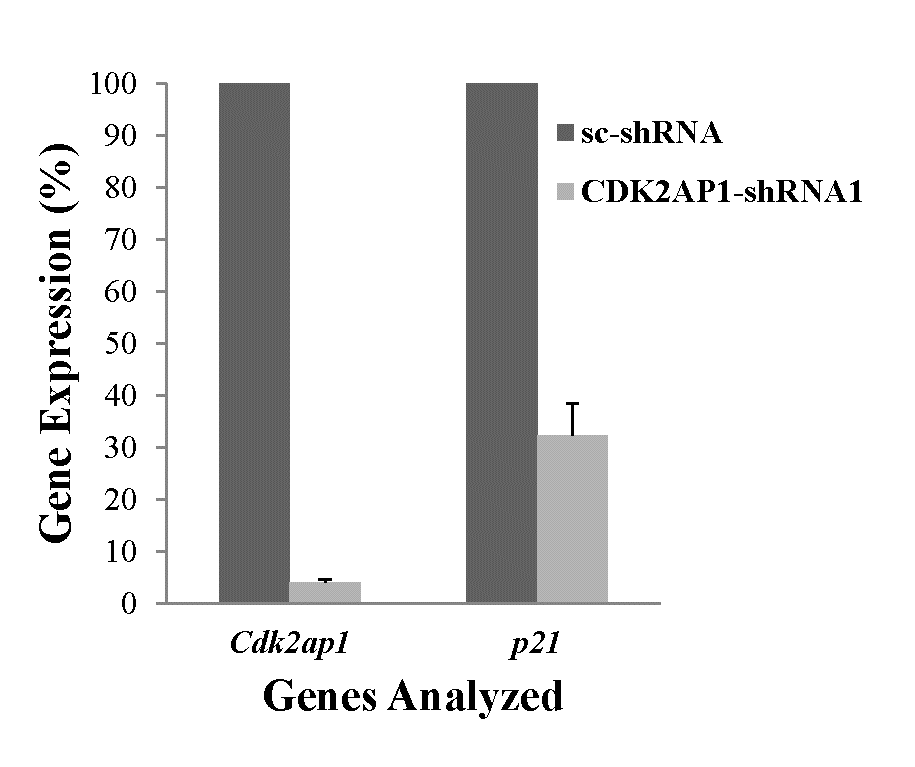

Supplement: S4 Fig — Quantitative PCR analysis showing the levels of CDK2AP1 and p21 expression in wild type and CDK2AP1 knockdown WA09 hESCs. Knockdown of CDK2AP1 resulted in a 63% reduction in p21 expression (p < 0.05. Comparisons were made between sc-shRNA and CDK2AP1-shRNA1 transduced cells for each gene analyzed). Results are presented together with standard deviation from experiments conducted in triplicate. (TIF) [file pone.0196817.s004.tif]

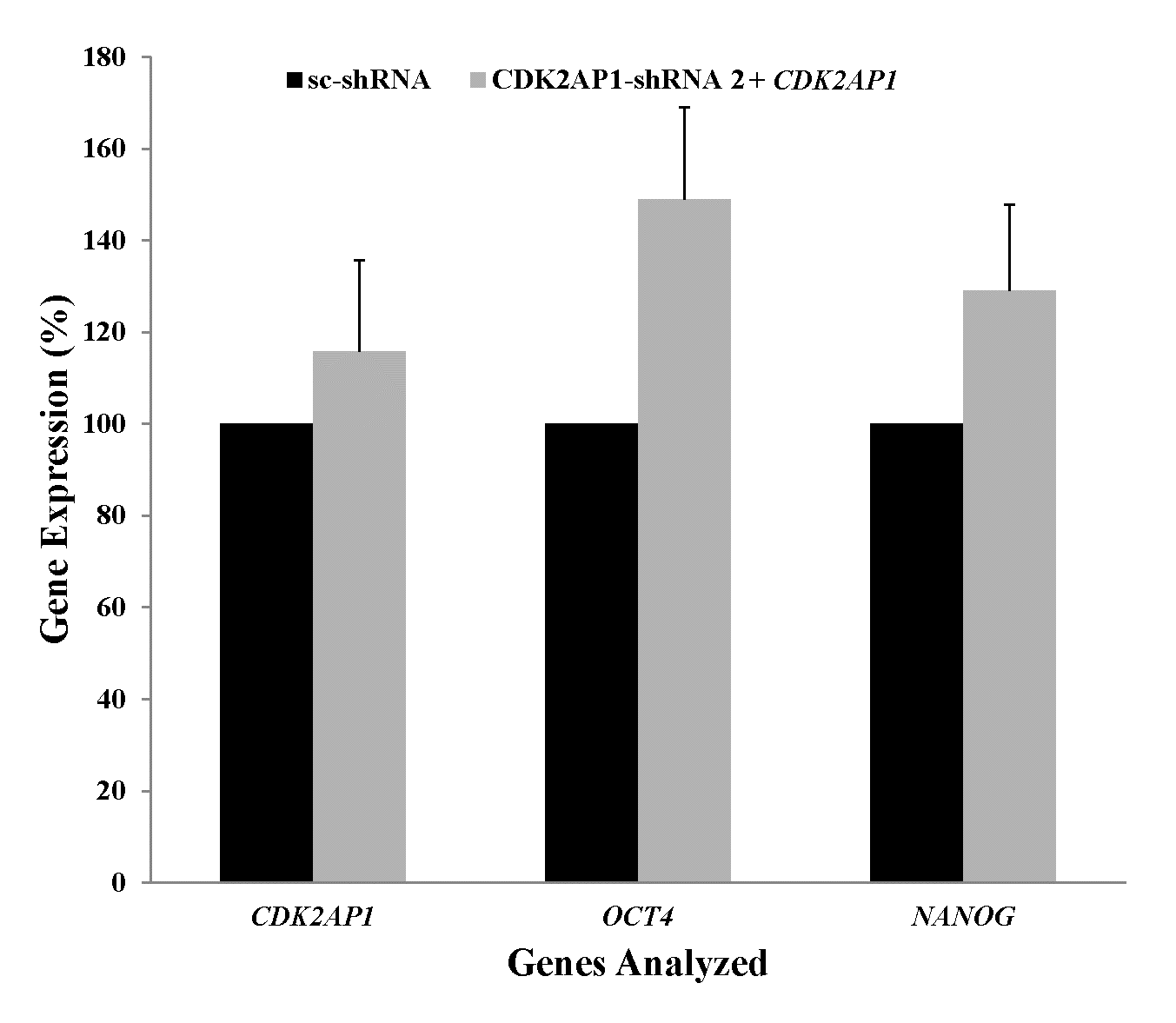

Supplement: S5 Fig — BG01v hESCs were transduced with sc-shRNA or with exogenous CDK2AP1 + CDK2AP1 shRNA2 and analyzed by qPCR for OCT4 and NANOG expression. Prevention of knockdown by introducing exogenous CDK2AP1 prevents the reduction in OCT4 and NANOG expression seen in CDK2AP1 knockdown hESCs (p> 0.05. Comparisons were made between sc-shRNA and CDK2AP1-shRNA2 + CDK2AP1 for each gene analyzed). Results are presented together with standard deviation from experiments conducted in triplicate. (TIF) [file pone.0196817.s005.tif]
