# Supplementary material for: Knockdown of CDK2AP1 in human embryonic stem cells reduces the threshold of differentiation
Source: PLoS One. 2018 May 7;13(5):e0196817. doi: 10.1371/journal.pone.0196817 (PMC5937771; doi:10.1371/journal.pone.0196817)
Supplement: S1 Table — (DOCX) [file pone.0196817.s006.docx]

**S1 Table: Sequences of primers used in qPCR analysis.**

| **Primer** | **Sequence** |
| --- | --- |
| GAPDH Forward | TTGCCATCAATGACCCCTTCA |
| GAPDH Reverse | CGCCCCACTTGATTTTGGA |
| CDK2AP1 Forward | ATGTCTTACAAACCGAACTTGGC |
| CDK2AP1 Reverse | GCCCGTAGTCACTGAGCAG |
| SOX1 Forward | ATGCACCGCTACGACATGG |
| SOX1 Reverse | CTCATGTAGCCCTGCGAGTTG |
| NESTIN Forward | CTGCTACCCTTGAGACACCTG |
| NESTIN Reverse | GGGCTCTGATCTCTGCATCTAC |
| T Brachyury Forward | TGCTTCCCTGAGACCCAGTT |
| T Brachyury Reverse | GATCACTTCTTTCCTTTGCATCAAG |
| IGF2 Forward | TCCTCCCTGGACAATCAGAC |
| IGF2 Reverse | AGAAGCACCAGCATCGACTT |
| AFP Forward | CTTTGGGCTGCTCGCTATGA |
| AFP Reverse | GCATGTTGATTTAACAAGCTGCT |
| GATA4 Forward | GTGTCCCAGACGTTCTCAGTC |
| GATA4 Reverse | GGGAGACGCATAGCCTTGT |
| CDKN1A (p21) Forward | TGTCCGTCAGAACCCATGC |
| CDKN1A (p21) Reverse | AAAGTCGAAGTTCCATCGCTC |
| Tp53 Forward | CAGCACATGACGGAGGTTGT |
| Tp53 Reverse | TCATCCAAATACTCCACACGC |
