# Supplementary material for: Knockdown of CDK2AP1 in human embryonic stem cells reduces the threshold of differentiation
Source: PLoS One. 2018 May 7;13(5):e0196817. doi: 10.1371/journal.pone.0196817 (PMC5937771; doi:10.1371/journal.pone.0196817)
Supplement: S2 Table — (DOCX) [file pone.0196817.s007.docx]

**S2 Table: List of antibodies and sources used in immunocytochemical and Western Blot analysis.**

| **Antibody** | **Catalog Number** | **Source** |
| --- | --- | --- |
| CDK2AP1 | sc-390283 | Santa Cruz Biotechnology |
| Phospho-Histone-3 | #3377 | Cell Signaling Technology |
| OCT4 | sc-101534 | Santa Cruz Biotechnology |
| NANOG | sc-293121 | Santa Cruz Biotechnology |
| Cyclin A1 | sc-751 | Santa Cruz Biotechnology |
| p53 | #9282 | Cell Signaling Technology |
